# Automatic Multi-Label ECG Classification with Category Imbalance and Cost-Sensitive Thresholding

**DOI:** 10.3390/bios11110453

**Published:** 2021-11-14

**Authors:** Yang Liu, Qince Li, Kuanquan Wang, Jun Liu, Runnan He, Yongfeng Yuan, Henggui Zhang

**Affiliations:** 1 School of Computer Science and Technology, Harbin Institute of Technology, Harbin 150001, China; 16B903062@stu.hit.edu.cn (Y.L.); wangkq@hit.edu.cn (K.W.); 20B903091@stu.hit.edu.cn (J.L.); yongfeng.yuan@hit.edu.cn (Y.Y.); 2 Peng Cheng Laboratory, Shenzhen 518000, China; hern@pcl.ac.cn; 3 School of Physics and Astronomy, The University of Manchester, Manchester M13 9PL, UK; 4 Key Laboratory of Medical Electrophysiology of Ministry of Education and Medical Electrophysiological Key Laboratory of Sichuan Province, Institute of Cardiovascular Research, Southwest Medical University, Luzhou 646000, China

**Keywords:** electrocardiogram, multi-label classification, deep neural network, category correlations, category imbalance

## Abstract

Automatic electrocardiogram (ECG) classification is a promising technology for the early screening and follow-up management of cardiovascular diseases. It is, by nature, a multi-label classification task owing to the coexistence of different kinds of diseases, and is challenging due to the large number of possible label combinations and the imbalance among categories. Furthermore, the task of multi-label ECG classification is cost-sensitive, a fact that has usually been ignored in previous studies on the development of the model. To address these problems, in this work, we propose a novel deep learning model–based learning framework and a thresholding method, namely category imbalance and cost-sensitive thresholding (CICST), to incorporate prior knowledge about classification costs and the characteristic of category imbalance in designing a multi-label ECG classifier. The learning framework combines a residual convolutional network with a class-wise attention mechanism. We evaluate our method with a cost-sensitive metric on multiple realistic datasets. The results show that CICST achieved a cost-sensitive metric score of 0.641 ± 0.009 in a 5-fold cross-validation, outperforming other commonly used thresholding methods, including rank-based thresholding, proportion-based thresholding, and fixed thresholding. This demonstrates that, by taking into account the category imbalance and predefined cost information, our approach is effective in improving the performance and practicability of multi-label ECG classification models.

## 1. Introduction

Cardiovascular disease has become the leading cause of death globally [1,2]. Electrocardiogram (ECG) monitoring is widely used for the screening and follow-up management of cardiovascular diseases. However, the interpretation of ECGs is a professional and time-consuming task. The rapid increase in ECG data has led to shortages in qualified physician resources. Consequently, the technology required for automatic ECG analysis and abnormality detection is in demand to cope with the rapid growth in ECG monitoring data [3]. Considering the diversity of cardiac conditions and the complex correlations between them, the automatic detection of ECG abnormalities can be formed as a multi-label classification (MLC) problem, i.e., multiple different kinds of abnormalities may coexist in a single ECG recording. An ideal ECG classifier, therefore, should identify all of the abnormalities existing in a recording.

The problem of multi-label ECG classification is challenging for several reasons. First, the number of label combinations that are possibly present in an ECG recording is very large. As there are more than 100 ECG labels, the label combinations amount to over 2^100^. Such an enormous number of combinations makes the learning task difficult to solve. Second, the categories needing to be classified are extremely imbalanced. In a realistic dataset, the samples of one category (e.g., sinus tachycardia) may be many orders of magnitude more than that of another category (e.g., Brugada syndrome). Furthermore, the problem regarding multi-label ECG classification is cost-sensitive, which means that the costs of misclassification between different pairs of categories can be different. The cost here refers mainly to the impact of the diagnosis on the patient’s prognosis: a diagnosis that may make a patient more unwell is costlier than one that may improve patient outcomes. For example, the cost of classifying an ECG recording of atrial fibrillation (AF) as atrial flutter (AFL) should be less than the cost of classifying an AF recording as normal sinus rhythm (NSR). As AFL is closer to AF than NSR in terms of outcomes and treatments, a prediction of AFL, although not accurate, may encourage doctors to further diagnose and treat patients to improve their condition, while a prediction of NSR would hide the disease and may lead to missing the optimum treatment time and exacerbating the disease.

MLC methods can be categorized into two groups: problem transformation methods and algorithm adaptation methods [4]. The concept of problem transformation methods is to transform the problem of multi-label classification into well-studied learning problems, such as binary classification and multi-class classification. Thus, the well-established methods used for the transformed problems can be used to solve the original problem. The most representative problem transformation methods include binary relevance [5], which decomposes MLC into a series of binary classification problems, and random k-label sets [6], which transforms MLC into a number of multi-class classification problems. Algorithm adaptation methods aim to adapt existing learning algorithms to tackle the MLC problem. Many traditional learning algorithms have been adapted for MLC along these lines, such as multi-label k-nearest neighbor (ML-kNN) [7], multi-label decision tree (ML-DT) [8], ranking-based support vector machine (rank-SVM) [9], and collective multi-label classifier (CML) [10]. Recently, deep learning algorithms have been widely adopted for MLC [11,12,13]. In particular, the method of binary relevance can be easily combined with deep learning models in a multi-task framework, where the detection of each label is regarded as a binary classification task, and a deep neural network (DNN) for representation learning is shared cross all binary classifiers [14]. As the representation learning is optimized for multiple tasks, the correlations among labels will be implicitly manifested in the intermediate representations, which can make up for a well-known drawback of binary relevance, i.e., the ignorance of label correlations. Due to its simplicity and efficiency, this method has been adopted in many studies for multi-label ECG classification [15,16,17]. However, the transformation to binary classifications further worsens the category imbalance, as the positive samples are generally much less than the negative samples for each binary classifier.

To address the category imbalance problem, a series of methods have been proposed generally following one of three approaches: data resampling, algorithm adaption, and ensemble learning. The data resampling techniques tackle the problem by removing instances of the most frequent classes (or majority classes) (i.e., undersampling) [18], or augmenting instances of the less frequent classes (or minority classes) (i.e., oversampling) [19]. Unlike traditional datasets where each sample has only one label, a sample in multi-label datasets may be associated with majority classes and minority classes simultaneously, which is known as concurrence among imbalanced labels [20]. If a dataset has a high concurrence between the majority and minority classes, the effectiveness of resampling would be reduced. The algorithm adaptation approach, on the other hand, adjusts the learning algorithms to make them suitable for the category imbalance problem. For example, the effect of category imbalance on the learning model can be alleviated by assigning different weights to the positive and negative samples of each label in loss calculation with methods such as weighted focal loss [21] and asymmetric loss [22]. The algorithm adaptation methods are usually tightly coupled with the adjusted algorithms, so their versatility will be reduced. The approach of ensemble learning is also commonly used to address the class imbalance problem in MLC. The basic idea is that the ensemble of multiple classifiers, each with a bias to different labels, will improve the model performance in learning imbalanced datasets. A series of ensemble techniques have been proposed in this approach, such as inverse random undersampling for the ensemble of binary classifiers [23] and the heterogeneous ensemble of multi-label classifiers [24]. Compared with previous methods, the ensemble learning approach is usually more computationally expensive, as multiple classifiers need to be trained and executed for prediction. The high computation complexity will hinder the algorithm from being embedded in platforms with limited computation resources, such as mobile ECG monitors.

The cost-sensitive property of multi-label ECG classification also has an important impact on the model performance, but has usually been ignored in previous studies. In a general sense, the methods for cost-sensitive learning can be classified into two categories: direct approaches and meta-learning approaches. The direct approaches adjust the learning algorithms by introducing cost information about misclassifications into the model training procedure, such as cost-sensitive decision trees [25] and cost-sensitive SVM [26]. By contrast, the meta-learning approaches do not adjust the learning algorithms, but modify the training data (pre-processing) or the model’s outputs (post-processing) to make the predictions cost-sensitive. One of the most representative pre-processing meta-learning methods is MetaCost, which relabels the training data according to pre-defined cost information. The post-processing methods are usually conducted by rescaling the model’s prediction or moving the thresholds based on the cost information [27]. One of the advantages of the post-processing methods is that the model predictions can be adjusted flexibly when the cost definitions are changed, while other methods must retrain the models to adapt to the changes of classification costs. However, the number of thresholds that need to be tuned is usually no less than the number of considered labels (perhaps several dozen or even hundreds), which results in the choice of proper thresholds being a challenging task in MLC. For example, if the number of thresholds is 20, and 10 candidate values are assessed for each threshold, the number of considered threshold combinations will be 10^20^, thus making a brute-force search infeasible.

In this study, we aim to develop a cost-sensitive learning framework for multi-label ECG classification. The developed framework is in the form of multi-task learning, where the stem part for representation learning is constructed with a deep residual network (ResNet) and class-wise attention (CWA), and the branches are multiple binary classifiers, with one for each category. In response to the challenges stated above, we propose a category imbalance and cost-sensitive thresholding (CICST) method to efficiently compute the thresholds for the binary classifiers according to predefined misclassification costs. The definition of “misclassification costs” considers not only the similarity of outcomes or treatments between categories, but also the category imbalance of the dataset. Furthermore, as the thresholding method is in a post-processing manner, the classification model can be adjusted flexibly to different cost definitions without retraining the model. This property allows our model to better adapt to application scenarios with different evaluation criteria.

## 2. Materials and Methods

In this section, we first introduce the datasets that were used for model training and evaluation in our study. We then present our method to address the problem of multi-label ECG classification. Our method consists of three parts, namely the pre-processing for data cleaning, the learning model based on a deep neural network (DNN), and the thresholding mechanism (i.e., CICST) to address the challenges of category imbalance and cost-sensitive learning.

### 2.1. Datasets

Several realistic datasets from the PhysioNet/CinC challenge 2021 named “*Will Two Do? Varying Dimensions in Electrocardiography*” [28] were used for model training and testing in this study. The dataset information is shown in Table 1. One of the data sources was the China Physiological Signal Challenge (CPSC) [29], which provided two datasets, namely CPSC and CPSC-Extra, in the list. The Physikalisch Technische Bundesanstalt (PTB) provided another two datasets, namely PTB and PTB-XL [30]. The dataset named G12EC was from the Georgia 12-lead ECG challenge. The last two datasets, namely Chapman-Shaoxing and Ningbo, were provided by the Chapman University and the Shaoxing People’s Hospital [31,32].

From the statistics of the datasets, we found some differences among these datasets. First, the number of recordings and label kinds are different from dataset to dataset. Furthermore, the number of label kinds in a dataset not proportional to the number of its recordings. For example, the dataset CPSC containing 6877 recordings involves nine kinds of labels in the annotations, while the dataset CPSC-Extra containing only 3453 recordings involves 72 kinds of labels (much more than that of CPSC). This indicates that the data distributions are quite different among these datasets, which may be attributed to the different data filtering or annotating rules among them. One example of the different data filtering rules is that each kind of label in CPSC have a relatively large sample size (≥236), while some labels in other datasets have very small sample sizes (as small as one). As for the annotating rules, the label granularity may be different from dataset to dataset. For example, incomplete right bundle branch block and complete right bundle branch block seem to be annotated as their supertype (i.e., right bundle branch block) in CPSC, while they are annotated distinctively in some other datasets. In addition, the recording lengths are different in some datasets, such as CPSC, CPSC-Extra, and PTB. In addition, the length ranges are also different among these datasets. Furthermore, the ECG recorder type and configurations may be different from dataset to dataset. For example, the device used by PTB is a non-commercial, PTB prototype recorder, while the device used by PTB-XL is from Schiller AG [30]. A consequence of this inconsistency is the diversity of sampling rate, ranging from 250 Hz to 1000 Hz. All these differences should be considered in the development of machine learning models.

### 2.2. Pre-Processing

Pre-processing is the first step in our learning framework. The purposes of pre-processing are to clean and normalize the ECG data. As the data are from different data sources, the sampling frequency and signal quality may be different from recording to recording. First, we resample the ECG signals to 250 Hz. The signals are then processed to remove baseline wanders and other kinds of noise. The baseline wander in signals is estimated by a moving the average filter in a window of one second (i.e., 250 sampling points). The estimated baseline is then subtracted from the original signals for baseline wander removal. The signals are further processed by a band-passing filter, with a passing band of 0.1~50 Hz, to suppress other kinds of noise, such as power-line interference, muscle noise, and respiration noise. Next, the signals are normalized to have mean zero and variance one. The difference in record lengths is another problem that should be addressed in the pre-processing stage. The DNN models are usually trained in mini-batches, where the records in a mini-batch should have the same signal length. For convenience in the model training, we convert the records in the training set to the same length by truncating (for longer records) or padding with zeros (for shorter records) from the end of the signals. The target length is set to 20 s in this work, as most of the records in the datasets are no longer than 20 s.

### 2.3. Structure of the Neural Network

**Figure 1:**
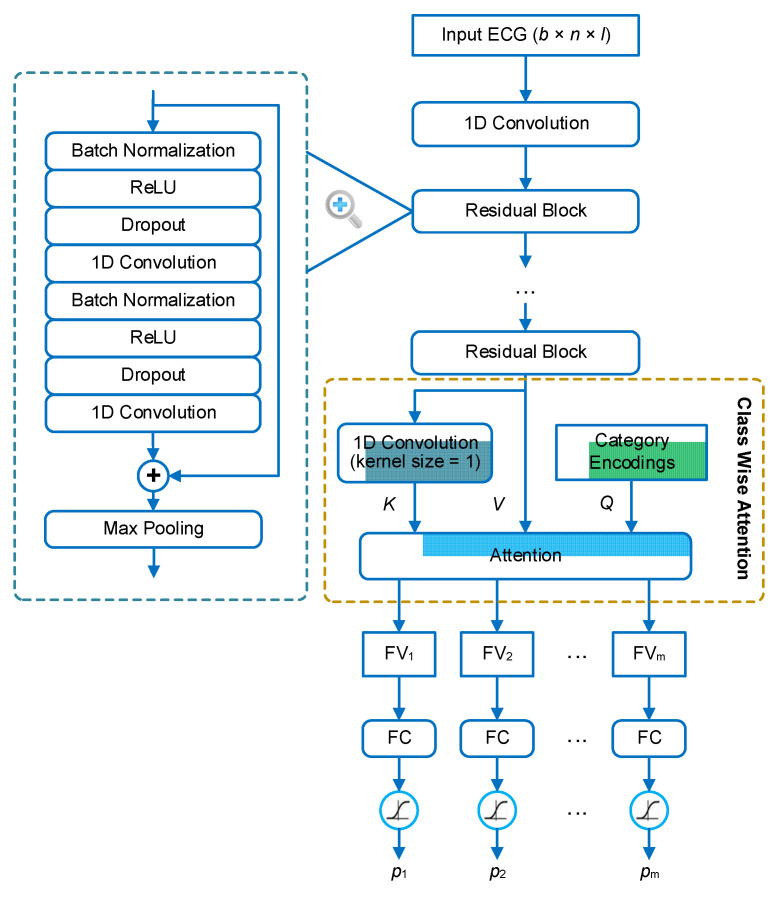
The architecture of our neural network for multi-label electrocardiogram (ECG) classification. The input of the network is an ECG signal in the shape of (*b* × *n* × *l*), where *b* is the batch size, *n* is the number of sampling points, and *l* is the number of ECG leads. The input ECG is first processed by a residual neural network to extract a feature map. The feature map is then processed by an attention layer to extract feature vectors. The inputs of the attention layer include keys (*K*), values (*V*), and queries (*Q*). The attention layer outputs a feature vector (denoted by FV_i_) for each category (indexed with *i*). *m* is the number of categories that are queried. FV_i_ is finally processed by a dedicated fully connected (FC) layer with sigmoid activation to generate the prediction for category *i* (denoted by *p*_i_).

We address the problem of multi-label ECG classification based on the methodology of deep learning. The architecture of the neural network is shown in Figure 1. The backbone of the network is a 1D residual neural network (1D ResNet), which consists of several residual blocks. The structure of each residual block is in a full pre-activation manner [33], where the first two layers in the block are batch normalization [34] and ReLU activation [35], respectively. In the residual block, the input is added to the output of the last convolutional layer through a short-cut connection. Thus, the main part of the block is aimed to learn a residual function between the output and the input. This structure has been demonstrated to be effective in facilitating the training of extreme deep neural networks [33,36]. The sum of the input and the residual is finally processed by a max-pooling layer for down-sampling. In our framework, the ResNet is used to extract features, or rather feature maps, from the ECG signals. The extracted feature map is a 3D tensor in the shape of *b* × *t* × *f*, where *b* is the batch size, *t* is the length in the time dimension, and *f* is the number of feature types.

In order to make classifications based on the extracted features, the feature maps are further aggregated to several feature vectors, one for each category. This aggregation is based on a mechanism, named class-wise attention (CWA), that was proposed in our previous study [21]. The idea behind CWA is that different categories may be associated with different time regions of the signal. For example, if premature atrial beats (PAB) and premature ventricular beats (PVB) coexist in an ECG recording, they are generally distributed in different heartbeats. Therefore, during the feature aggregation, the detector of PAB expects the features of the PABs to be present in the feature vector, while the detector of PVB expects the features of the PVBs to be present in the feature vector. If all detectors use the same feature vector, there will be a competition between the features of different categories, and the features of some categories (i.e., categories with subtle features or small sample sizes) may be at a disadvantage. To address this problem, CWA tries to search relevant features for each detector based on the attention mechanism, and aggregate the feature maps according to the degrees of relevance. In CWA, the inner attention layer has three inputs: the query vectors (*Q*), the key vectors (*K*), and the value vectors (*V*). *Q* includes the encodings (one-hot encoding in our implementation) of the categories. *K* is transformed from the feature maps based on a convolutional layer with the kernel size equal to 1 and the filter number equal to the category number. *V* contains just the feature maps. The output of CWA contains the feature vector with respect to each query vector (or category). Finally, each feature vector is input into a dedicated fully connected (FC) layer to predict the probability that the corresponding category is present in the recording.

### 2.4. Class Imbalance and Cost-Sensitive Thresholding

The outputs of the model on a sample are a list of scalar values in the range of 0–1 that specify the confidence that the sample belongs to each considered category. To generate categorical predictions, a threshold is generally applied on the scalar values. The choice of threshold will affect the trade-offs between positive and negative errors. Raising the threshold will reduce the chance that a sample is classified as positive, as the criteria for positive is more stringent. By contrast, the increased threshold will lead to more samples to be predicted as negative, as the number of negative predictions and the number of positive predictions are negatively related. As the ECG classification is cost-sensitive, the choice of thresholds should minimize the expected classification costs on the test set. The mechanism of CICST consists of two parts: one is the method for obtaining the cost information of different kinds of misclassification, and the other is the method for calculating the threshold for each binary classifier based on the cost information.

#### 2.4.1. Obtaining the Cost Matrix

The cost of misclassifying one category for another can be assessed in different ways. In this study, we consider the misclassification cost from two perspectives. The first is the similarity of outcomes and treatments between categories. In other words, if two categories of ECG have similar clinical risks or should be treated similarly, the cost of misclassification between them would be relatively small. The other perspective is the ratio of frequencies between categories in the training dataset. It has been demonstrated that the frequency ratio between categories can affect the distribution of classifiers’ outputs [27]. In our learning framework, the categories (positive or negative) faced by most binary classifiers are extremely imbalanced, where there are much less positives samples than negative samples. As a consequence, the learning algorithm tends to classify a large proportion of positive samples into the negative category. From the application point of view, the positive class is actually the focus of the classification problem. The cost of a false positive detection would be conducting additional tests, while the cost of a false negative detection would be the severity of an illness. Therefore, the property of category imbalance and the significance of the positive categories should also be taken into account in the definition of classification costs. Furthermore, the classification costs need to be quantified for comparison and calculation purposes. Ideally, the cost of misclassification between each pair of categories should be specified. The costs are generally organized in a cost matrix, denoted by C. The entry C[i,j] specifies the cost of classifying a sample as category *i* when the true category is *j*.

The measurement of classification costs is a highly skilled and delicate task. Thanks to the organizers of the PhysioNet/CinC challenge 2020/2021 [28,37], a benefit matrix judged by the cardiologists of the challenge is now available. The benefit matrix, denoted by B, contains information on the relationships of the 26 ECG categories. The entry B[i,j]∈[0,1] specifies the benefit of classifying a sample belonging to category *j* as a category *i*. A high value of B[i,j] indicates that categories *i* and *j* have similar outcomes or treatments, and vice versa. B[i,j] reaches its maximum value (i.e., 1) when *i* is equal to *j*. The details of this benefit matrix are available at https://github.com/physionetchallenges/evaluation-2021 (accessed on 30 September 2021). The benefit matrix can be regarded as the opposite of the cost matrix; thus we can derive a cost matrix from the benefit matrix with the formula: C′=1−B.

As the multi-label classification is converted to multiple binary classification problems, we need the cost information of false negatives and false positives to calculate the threshold for detecting each category. To address this problem, we define the false negative cost for each category to be 1, and calculate the false positive costs, denoted by c′, based on the cost matrix C′ and the label distribution in the training set. The algorithm for converting the cost matrix C′ to the false positive cost of each category is shown in Algorithm 1. The input of the algorithm include the cost matrix C′ and the label matrix $Y\in {\left\{0,1\right\}}^{N\times m}$ of the training set. The entry Y[i,j]=1, if and only if the sample *i* is annotated with label *j*. In this algorithm, Y is first normalized to $Y^{n}$ by dividing each entry by the annotated label number of its corresponding sample. To avoid dividing by zero, the entries of samples with no labels are divided by 1. The misclassification cost of each label on each sample is then calculated by the dot product between the normalized label matrix and the cost matrix. The resulting entries with positive labels are multiplied by zero, as the cost of a right prediction is zero. Finally, the false positive costs for each category are averaged over all samples to obtain the representative false positive costs, denoted by c′.
**Algorithm 1** The pseudo-code of the algorithm to convert the cost matrix to multi-label classification cost. *C*′ denotes the cost matrix specifying the misclassification cost between each pair of categories. *Y* is the label matrix of the training dataset. ***c***′ contains the derived false positive costs for each categoryc′**= computeFalsePositiveCosts (**C′**,**Y**)**# *Normalize the training set labels* Y *to* $Y^{n}$**for** *i* = 1 **to** *N* **do**# *Calculate the label number of samples i*L[i]= max(sum(Y[i,1:m]), 1)# *Normalize the training set labels* Y$Y^{n}\left[i,\;1:m]=Y[i,\;1:m\right]/L\left[i\right]$**endfor**# *Calculate the misclassification cost of each label on each sample*$SC^{\prime }=$ matrix_multiply ($Y^{n}$, C′)# *Mask out the entries with positive labels*$SC^{\prime \prime }=SC^{\prime }\cdot \left(1-Y\right)$# *Calculate the mean false positive costs of each label in the training set***for** *i* =1 **to** *m* **do**$c^{\prime }\left[i\right]$ = $\mathrm{sum}\left(SC^{\prime \prime }\left[1:N,\;i\right]\right)/\mathrm{sum}\left(1-Y\left[1:N,i\right]\right)$**endfor**Return $c^{\prime }$

The false positive cost vector c′ does not reflect the property of class imbalance. Therefore, we designed a method to merge the cost information from both a prior cost definition and the imbalance ratios (IRs). The IR is defined as the ratio of the negative sample number to the positive sample number in the training set. The false positive cost of label *i* is then defined as follows:

(1) $$c_{i}=\frac{{c\prime }_{i}^{\alpha }}{IR_{i}^{1-\alpha }}\;$$

where α∈[0,1] is a modulating factor. When α=0, $c_{i}=c\prime_{i}$ ignoring the effects of category imbalance on the model performance. When α=1, $c_{i}=IR_{i}^{-1}$ ignoring the effects of predefined misclassification costs with respect to the category similarities. Therefore, to ensure that the final cost definition involves both kinds of information, the value of α should be between 0 and 1. In the results section, we will evaluate the influence of different choices of α on the model performance.

#### 2.4.2. Calculation of Thresholds

With the cost information obtained, we need to compute the threshold for each binary classifier in our framework. The method to calculate the threshold for a binary classifier based on the cost information has been proposed in previous studies [27]. With the same method, we can derive the thresholds from the estimated costs as follows:

(2) $$t_{i}=\frac{CM_{i}\left[1,0\right]-CM_{i}\left[0,0\right]}{CM_{i}\left[1,0\right]-CM_{i}\left[0,0\right]+CM_{i}\left[0,1\right]-CM_{i}\left[1,1\right]}=\frac{c_{i}}{1+c_{i}}\;$$

where $CM_{i}\left[0,0\right]$ and $CM_{i}\left[1,1\right]$ denote the cost of true negative and true positive predictions for category *i*, respectively. They are all zero in our definition of the costs. $CM_{i}\left[1,0\right]$ denotes the cost of a false positive prediction for category *i*, thus $CM_{i}\left[1,0\right]=c_{i}$. Analogously, $CM_{i}\left[0,1\right]$ denotes the cost of a false negative prediction for category *i*, which is 1 in our definition. If the model prediction for category *i* on a sample is larger than the threshold $t_{i}$, category *i* will be added to the predicted label set of the sample.

### 2.5. Experimental Setup

The learning framework is implemented with the TensorFlow framework. To better evaluate the effects of the thresholding methods, we use binary cross-entropy without sample or category weighting as the loss function for model training. The Adaptive Moment Estimation (Adam) method (with β_1_ = 0.9 and β_2_ = 0.999) is used to update the model parameters during the model training. The initial learning rate is 0.001, then it is scheduled with the exponential decay method with the decay factor equal to 0.9.

We train and evaluate the learning framework in a 5-fold cross-validation manner. The original datasets are first shuffled randomly, and then evenly split into 5 subsets. In each fold, 4 subsets (including about 70,500 recordings) are used as the training set, and the rest (including about 17,600 recordings) is used as the validation set. We evaluate the model performance on the validation set of each fold.

### 2.6. Metrics

In view of the cost-sensitive characteristic of multi-label ECG classification, we adopt a scoring metric recently proposed by the PhysioNet/CinC challenge [28] to measure the model performance. We name this metric cost-weighted accuracy (*CWAcc*) as it is derived by introducing cost-based weights to the traditional accuracy metric. To calculate *CWAcc*, the prediction results on the validation set should be organized in a multi-class confusion matrix, denoted by $A=\left[a_{i,j}\right]$, where $a_{i,j}$ is the number of recordings of category *j* classified to category *i*. The score is computed by weighted averaging the matrix *A*:

(3) $$CWAcc=\underset{i,j}{\sum }a_{i,j}b_{i,j}$$

where $b_{i,j}$ is the entry of the benefit matrix *B* that measures the similarity between each pair of categories in terms of outcomes and treatments. Furthermore, *CWAcc* is normalized so that a score of 1 is obtained when the classifier always outputs the right labels, and a score of 0 is obtained when the classifier always outputs the normal class.

We also calculate some other commonly used metrics in clinical scenarios to evaluate the performance of our method, including accuracy (*Acc*), sensitivity (*Se*), and specificity (*Sp*). The *Acc* is defined as the proportion of samples that are correctly classified for each of the considered labels:

(4) $$Acc=\frac{1}{N}\overset{N}{\underset{i=1}{\sum }}1_{Y\left[i,:\right]=\hat{Y}\left[i,:\right]}$$

where *N* denotes the number of test recordings, Y[i,:] denotes the annotated labels of recording *i*, and $\hat{Y}\left[i,:\right]$ denotes the predicted labels of recording *i*. $1_{Y\left[i,:\right]=\hat{Y}\left[i,:\right]}$ is the indicator function, which is one if the predicted labels for recording *i* are same as the annotated labels, and zero otherwise. To calculate the *Se* and *Sp*, we first calculate the overall true positives (*TP*), false positives (*FP*), true negatives (*TN*), and false negatives (*FN*) by summing the corresponding category-wise statistics over all considered categories: $TP=\sum_{i}^{m}TP_{i}$, $FP=\sum_{i}^{m}FP_{i}$, $TN=\sum_{i}^{m}TN_{i}$, $FN=\sum_{i}^{m}FN_{i}$, where *m* is the number of categories. The *Se* and *Sp* are then calculated as follows:

(5) $$Se=\frac{TP}{TP+FN}$$

(6) $$Sp=\frac{TN}{TN+FP}$$

## 3. Results

To evaluate the methods proposed above, we conducted a series of experiments. In this section, we first introduce the experiment setups and the evaluation metrics. We then describe the results to assess the model performance and to make a comparison with other methods.

### 3.1. Effects of α Values

**Figure 2:**
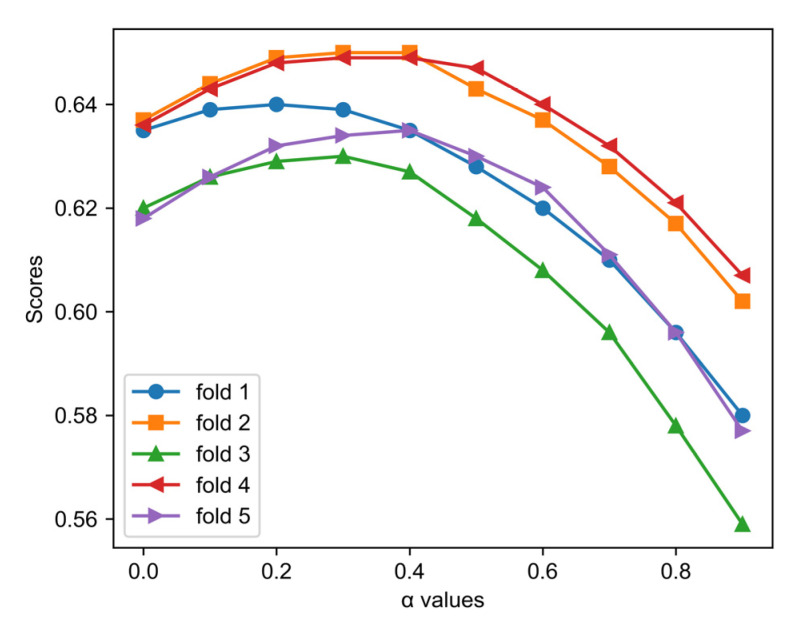
The cost-weighted accuracy (*CWAcc*) scores of the model under different values of α. Each line in this figure corresponds to a model trained in a certain cross-validation fold.

The parameter α controls the balance between the category imbalance-related costs and the pre-defined costs with regard to the similarities among categories. To evaluate the effects of α values on the results of the thresholding, we calculated the metric scores on the validation sets under different α values. The results are shown in Figure 2. It is clear that, in each cross-validation fold, the choice of α value has an important impact on the model performance. As the α value increases from 0 to 1, the metric scores gradually rise and level off, then gradually fall again. This changing pattern is very similar among the cross-validation folds, indicating that the influence of α on the model performance has a stable regularity. The maxima are obtained when α is in the range of 0.2–0.4, with slight variations among the cross-validations. Hence, we set α to 0.3 in the following experiments.

### 3.2. Comparisons with Other Thresholding Methods

In the experiments, we also compared the performance of CICST to other commonly used thresholding methods, including rank-based thresholding (Rcut), proportion-based thresholding (Pcut), and fixed thresholding (Fixed) [38]. The Rcut strategy sorts the labels by the predicted scores and assigns *t*-top ranking labels to each sample. Usually, *t* is set to the label cardinality of the training set, i.e., the average number of labels per training sample. In our training set, the label cardinality is 2, thus *t* is set to 2 in our experiments. The Pcut method sorts the test samples by score and classifies $k_{j}$ top-ranking samples as positive for category *j*. The $k_{j}$ is usually set to the proportion of the category *j* in the training set. The fixed thresholding method may be the most prevailing thresholding method, assigning a fixed threshold (usually 0.5) to all categories. In our experiments, other fixed threshold choices are also examined, and the results obtained with a threshold of 0.2 are reported for its superior performance.

The results of the different thresholding methods are shown in Table 2. CICST achieved the highest *Se* (0.823 ± 0.012) and *CWAcc* (0.644 ± 0.009) among all the tested thresholding methods. In addition, it outperformed Rcut, Pcut, and fixed thresholding with a threshold of 0.5 on both of the metrics by a large margin. The scores of fixed thresholding on *Se* and *CWAcc* were also lower than those of CICST, but the differences were smaller. The results also show that methods with a higher *Se* tend to have a higher *CWAcc*, but also tend to have a lower *Sp*. The *Sp* of CICST was lowest among all these methods. Fixed thresholding with a threshold of 0.5 achieved the highest *Sp*, but its *Se* and *CWAcc* were lowest and second lowest, respectively. The antagonism between *Se* and *Sp* is because moving the threshold has the opposite effect on *TP* and *TN*. The positive correlation between *Se* and *CWAcc* stems from the fact that false negatives are usually more costly than false positives in disease diagnosis. By contrast, *Acc* was not positively correlated with *CWAcc*, reflecting that whether or not classification losses are considered will lead to significant differences in model assessments.

### 3.3. Comparisons with Public Results of the Challenge

To evaluate the performance level of the proposed method, we further compared our results with the public state-of-the-art results in the PhsioNet/CinC challenge 2021. As the public results are calculated on the hidden test set of the challenge, it is unfair to compare them with our 5-fold cross-validation results. The hidden test set consists of four subsets, including a hidden CPSC test set, a hidden G12EC test set, an undisclosed American dataset, and an UMich dataset from the University of Michigan. Among these subsets, we know that the training set and test set in each of CPSC and G12EC are approximately independent and identically distributed. Therefore, if we exclude one of the training sets from the training process and evaluate the model on it, the results should be comparable, although not exactly, to the results on the corresponding test set. Specifically, for each of the CPSC and G12EC training sets, we first split it into five subsets. Among these subsets, one is used for model evaluation, and the remaining four subsets and other datasets are used for model training. We let the five subsets take turns acting as the validation set to implement a modified version of cross-validation. In this way, we obtain the validation results of our method on the training sets of CPSC and G12EC.

The comparison of our results and the public results, involving the top 10 teams in the challenge, are shown in Table 3. Note that the rank in the table is based on the overall test results on all of the hidden test sets, rather than on the scores shown in the table. It can be found that high scores on the test sets of CPSC and G12EC does not necessary lead to a high rank in the overall results. For example, the team CeZIS achieved the highest *CWAcc* on both test sets of CPSC and G12EC, but ranked only sixth in the overall results. This can be attributed to the diversity of data distributions among the test datasets. In the modified local 5-fold cross-validation, our method achieved a *CWAcc* of 0.723 ± 0.010 on the CPSC training set, and a *CWAcc* of 0.580 ± 0.006 on the G12EC training set. Our model scores are not the best ones, but they are in the score range of the top 10 teams of the challenge. In addition to thresholding, many factors will affect the performance of a classifier, such as data pre-processing, model architecture, hyperparameters, and so on. The lower amount of data (4/5 of the original training set) used in the training of our model may also result in a lower score for the model.

### 3.4. Adaptation to Changes of Cost Deffinition

**Figure 3:**
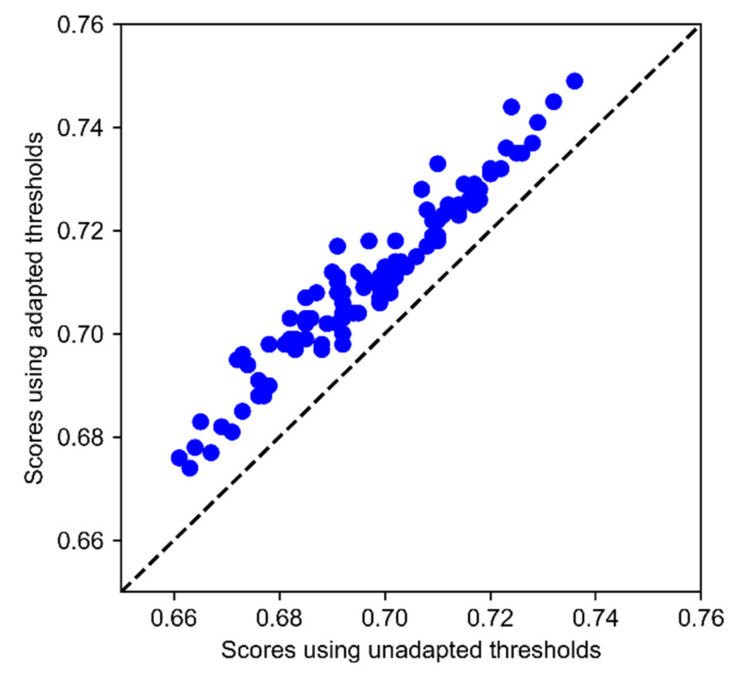
The comparison between scores with and without adaptation to new cost definitions in 100 simulated cost changes. The *x*-axis indicates the scores that are achieved by CICST using the original cost definitions, while the *y*-axis indicates the scores that are achieved by CICST using the updated cost definitions. Each solid circle in the figure represents a simulated cost change.

A potential advantage of CICST is that it can adapt to a variation of cost definitions. To evaluate this potential advantage, we conducted an experiment by simulating the cost changes. To simulate a cost modification, a proportion of categories are randomly selected, and the entries of the benefit matrix relevant to the selected categories are increased or decreased by a certain percentage. We then use the new benefit matrix to evaluate the previously trained models. As CICST uses the information from the benefit matrix in computing the thresholds, it can update the thresholds according to the new benefit matrix. We wanted to check whether the model would get a performance boost from updating the thresholds. Specifically, in a simulation, 50% of the categories are randomly selected, and their relevant entries in the benefit matrix are increased by 50%, but the entries on the diagonal remain unchanged. This simulation is repeated 100 times, each with different categories selected. The results are shown in Figure 3. From this figure, we can see that all the solid circles are above the dashed line. This indicates that the classifier achieves higher performance by updating the thresholds with CICST according to the changed cost information.

**Figure 4:**
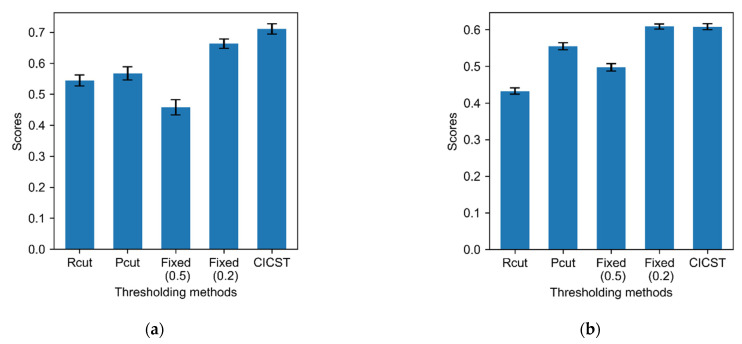
Comparisons of the scores achieved by different thresholding methods after the costs are changed. (**a**) Scores of different thresholding methods after 50% of the entries of the benefit matrix are increased by 50%; (**b**) Scores of different thresholding methods after 50% of the entries of the benefit matrix are decreased to 70% of the original values.

We also evaluated the performance of other thresholding methods under the changed costs, with the results shown in Figure 4. The left figure shows the model performances after the entries relevant to the randomly selected categories are increased by 50%, while the right figure shows the model performances after the relevant entries are decreased to 70% of their original values. In the case of the increasing entries, CICST achieves the best results among all the methods. In the case of the decreasing entries, the performance of CICST is also optimal among the tested methods, but is very close to that of the fixed threshold of 0.2. On the whole, the results indicate that CICST has a better adaptation ability to the change classification costs.

### 3.5. Instance Analysis of the Predictions

**Figure 5:**
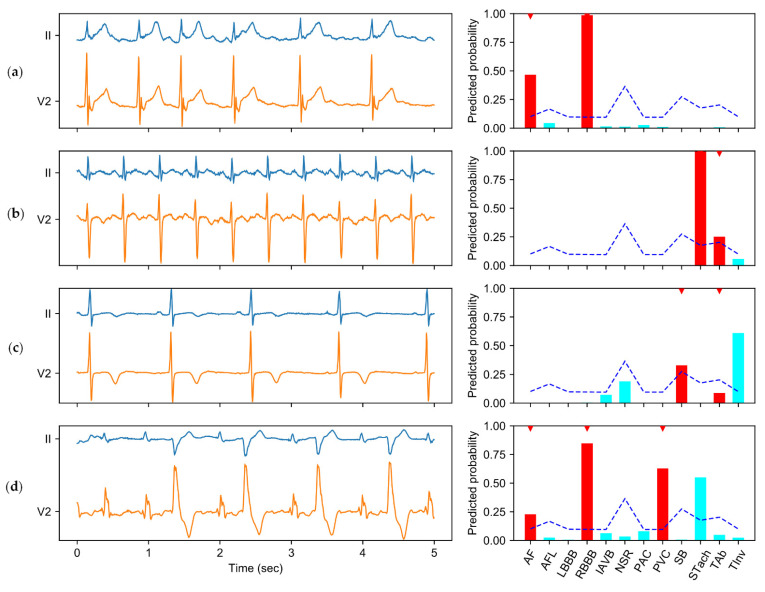
Instances of ECG recording and model predictions. The subgraph on the left of each row shows the ECG signal (including lead II and lead V2), while the subgraph on the right shows the model predictions for the left ECG signal. The predicted probabilities for the physician-annotated labels are in red bars and marked with red downward triangles above the bars, while the predictions for other labels are shown in the cyan bars. The thresholds calculated by CICST are shown in blue dashed lines. Four instances are shown in this figure: (**a**) an ECG recording with annotated labels of atrial fibrillation (AF) and right bundle branch block (RBBB); (**b**) an ECG recording with labels of sinus tachycardia (STach) and T-wave abnormal (TAb); (**c**) an ECG recording with labels of sinus bradycardia (SB) and TAb; and (**d**) an ECG recording with labels of AF, RBBB, and premature ventricular contraction (PVC). This figure also shows the predicted probabilities for other labels, including atrial flutter (AFL), left bundle branch block (LBBB), first-degree atrioventricular block (IAVB), normal sinus rhythm (NSR), premature atrial contraction (PAC), and T-wave inversion (TInv).

To better illustrate how CICST works in multi-label ECG classification, we show some examples in Figure 5. Figure 5a shows an ECG recording with physician-annotated labels of atrial fibrillation (AF) and right bundle branch block (RBBB). According to the thresholds calculated by CICST (shown in the blue dashed line), the predicted labels are consistent with the annotated labels in this example. Similarly, in Figure 5b, CICST also enables the model to correctly classify a recording with sinus tachycardia (STach) and T-wave abnormal (TAb). In Figure 5c, the model with CICST made a true positive classification of sinus bradycardia (SB), a false negative classification of TAb, and a false positive classification of T-wave inversion (TInv). However, the ECG waveform does show characteristics of TInv. Therefore, it is likely that the annotator missed the label of TInv, but the model detected the label based on the rules learned from the training data. In Figure 5d, the model correctly detected AF, RBBB, and premature ventricular contraction (PVC), but also made a false positive detection of STach. The false detection shows that the model established a correlation between elevated heart rate and STach, but failed to distinguish sinus and non-sinus tachycardia effectively. In these instances, the threshold calculated by CICST was effective to increase the sensitivity of the classifier to some diseases, e.g., TAb in Figure 5b and AF in Figure 5d. Moreover, the high threshold for normal sinus rhythm (NSR) helps to avoid false positive predictions of NSR, as shown in Figure 5c.

## 4. Discussion

This work proposes an approach to achieving cost-sensitive multi-label ECG classification using a thresholding method, namely CICST. It has been found that medical diagnosis is a cost-sensitive problem [26]. However, this property has been widely ignored in previous studies on ECG classification methods. Most of the previous studies have focused on designing novel methods to improve the accuracy of the classifier. Some studies have tried to strike a balance between sensitivity and specificity, particularly in cases of category imbalance [15,21]. As we cannot achieve a perfect algorithm that makes correct predictions in every instance, the domain-related classification costs help us to find a reasonable balance when optimizing the algorithm. If the cost-sensitive property is not considered, the optimization goal of the algorithm would be mismatched with the actual demand.

The experimental results demonstrate the effectiveness of CICST in addressing the problem of multi-label ECG classification. Although all of the tested thresholding methods share the sample classification model, different choices of thresholds have substantial impacts on the model performance. In particular, the fixed threshold of 0.5 is used as the default threshold for a wide spectrum of classifiers. However, due to the category imbalance, this default thresholding strategy suffers a severe performance slump. By contrast, CICST is effective in finding proper thresholds to address the challenge of extreme category imbalance. Moreover, the performance of fixed thresholding with the threshold of 0.2 is close to that of CICST. However, a proper fixed threshold value should be tuned on the validation set, while CICST computes the threshold directly from the cost definition and the imbalance ratios.

As CICST is a post-processing method, it can be applied to a series of existing classification algorithms without the need to modify the original algorithms. Furthermore, as demonstrated in the experiments, CICST is flexible to adapt to changes in the definition of classification costs. This ability has important practical implications. It makes it easy to customize a classifier with a particular purpose. For example, by changing the definition of the cost or benefit matrix and then applying the CICST method, we can increase the sensitivity of the classifier to some categories, while increasing the specificity to other categories without retraining the model.

This work also has some limitations. First, the cost information in the cost matrix is converted to the costs for binary classification, which may lead to a certain loss of cost information. Second, the category relationships are explored implicitly in a multi-task learning framework. However, this cannot avoid the presence of unreasonable predictions, e.g., normal sinus rhythm and some kinds of arrhythmias may be predicted to coexist in a recording. Third, the possible differences in annotation rules among different datasets are not addressed in this study. Furthermore, the lack of interpretability of deep learning models may be a hindrance to clinical application. How to improve the model’s interpretability is an open question with significant implications. Therefore, more sophisticated methods for utilizing the cost information should be explored in further research, and new mechanisms should also be studied to explicitly utilize the label relationships, handle the diversity of the annotation rules, and improve the interpretability of the predictions.

## 5. Conclusions

In this study, we proposed a deep-learning framework and a thresholding method for multi-label ECG classification. We evaluated our method against datasets from the PhysioNet/CinC challenge 2021, and the achieved *CWAcc* score in a 5-fold cross-validation was 0.641 ± 0.009, outperforming other commonly used thresholding methods. The experimental results demonstrated the effectiveness of our method in addressing the challenges of class imbalance and the cost sensitivity of multi-label ECG classification. In particular, the thresholding method (i.e., CICST) can enable the classifier to adapt to a variation in cost definition without the need for retraining. Therefore, our method has great potential to be applied in a wide spectrum of ECG analysis scenarios, and may also have implications for other fields of multi-label learning.

## Figures and Tables

**Table 1:** Information of the datasets used in this study.

Datasets	No. of Recordings	No. of Label Kinds	Recording Lengths
CPSC	6877	9	6~144 s
CPSC-Extra	3453	72	8~98 s
PTB	516	17	32~120 s
PTB-XL	21,837	50	10 s
G12EC	10,344	67	5~10 s
Chapman-Shaoxing	10,247	54	10 s
Ningbo	34,905	80	10 s

**Table 2:** The metric scores (mean ± std) achieved by different thresholding methods in 5-fold cross-validations.

Thresholding	Accuracy	Sensitivity	Specificity	*CWAcc* ^1^
Rcut	0.123 ± 0.003	0.729 ± 0.004	0.962 ± 0.000	0.469 ± 0.005
Pcut	0.493 ± 0.012	0.695 ± 0.005	0.982 ± 0.001	0.556 ± 0.009
Fixed (0.5)	**0.520 ± 0.008**	0.629 ± 0.022	**0.989 ± 0.001**	0.493 ± 0.030
Fixed (0.2)	0.436 ± 0.012	0.786 ± 0.013	0.971 ± 0.001	0.635 ± 0.011
CICST	0.387 ± 0.016	**0.823** ± 0.012	0.957 ± 0.003	**0.641** ± 0.009

^1^ *CWAcc* is short for cost-weighted accuracy.

**Table 3:** The public results on the China Physiological Signal Challenge (CPSC) and the Georgia 12-lead ECG challenge (G12EC) test sets and the modified 5-fold cross-validation results (mean ± std) of our method on the training sets of CPSC and G12EC.

Rank in the Challenge ^1^	Team Name	CPSC		G12EC	
		Accuracy	*CWAcc*	Accuracy	*CWAcc*
1	ISIBrno-AIMI	0.422	0.725	0.250	0.617
2	NIMA	0.591	0.772	0.253	0.620
3	DSAIL_SNU	0.258	0.640	0.179	0.594
4	Cardiocallenger	0.405	0.699	0.268	0.619
5	USST_Med	0.252	0.574	0.179	0.597
6	CeZIS	**0.650**	**0.910**	0.286	**0.673**
7	SMS + 1	0.370	0.638	0.175	0.554
8	DataLA_NUS	0.455	0.650	0.307	0.630
9	Dr_Cubic	0.585	0.845	0.280	0.637
10	Ami_kagoshima	0.499	0.763	**0.350**	0.600
-	Ours	0.633 ± 0.013	0.723 ± 0.010	0.194 ± 0.004	0.580 ± 0.006

^1^ The rankings are based on the cost-weighted accuracy (*CWAcc*) score calculated on all of the four hidden test sets. Detailed public results can be found at https://physionetchallenges.org/2021/leaderboard/ (accessed on 1 November 2021).

## Data Availability

The datasets used in this study are available at https://physionetchallenges.org/2021/ (accessed on 1 September 2021).
